# Nonoperative hip fracture care: how conservative should we be?

**DOI:** 10.2340/17453674.2025.43190

**Published:** 2025-02-24

**Authors:** Antony JOHANSEN, Bjarke VIBERG

**Affiliations:** 1 University Hospital of Wales, Trauma Unit, Cardiff, UK; 2 OUH, Department of Orthopaedic Surgery and Traumatology, Odense University Hospital, Odense C, Denmark

The mental and physical frailty of the people who typically sustain a hip fracture means that for a quarter this injury will mark the beginning of the last year of their life. Clinical pathways and care decisions must therefore be sensitive to their priorities for the end of their life.

The study by Zeelenberg et al. [1], recently published in *Acta Orthopaedica*, includes hip fracture patients with a very poor life expectancy, from 2 separate cohorts who are treated nonoperatively after shared decision-making. The first cohort (n = 88) includes hip fracture patients admitted to hospital but then managed nonoperatively, and nearly half then discharged to a nursing home. The second cohort (n = 20) had been assessed for a suspected hip fracture in their nursing home but were not sent to hospital. The median survival time was 7 days in the hospitalized cohort and 5 days in the non-hospitalized cohort.

In both cohorts, pain levels were felt to be acceptable, and treatment satisfaction and quality-of-dying as rated by proxies or caregivers was very high. The study has answered the question of whether nonoperative care can provide good symptom control and end-of-life care to patients with hip fracture. But what questions does it raise?

## Can we identify people in whom hip fracture is very likely to lead to death?

The ready availability of hip fracture data means that the literature now includes huge numbers of study models to predict mortality. A recent meta-analysis by Bui et al. found a high American Society of Anesthesiologists (ASA) grade to be the strongest single preoperative predictor of risk, with an ASA grade of 3+ carrying an odds ratio of 2.69 for 30-day mortality [2].

Despite this, a study based on National Hip Fracture Database (NHFD) data for 59,369 patients [3] showed that the 0.5% of patients who were in the highest risk category (ASA grade 5: defined as “a moribund patient who is not expected to survive without operation”), actually had a 20-day mortality of just 20%, and that two-thirds survived to leave hospital.

Many prediction tools may be statistically useful at a population level, but they cannot do so reliably for an individual patient. The study by Zeelenberg et al. appeared to describe excellent preoperative assessments, but even so the clinical teams could not be certain of the prognosis for the patients whose management options they were discussing [1]. The fact that most died within a week of nonoperative management cannot be viewed as proof of correct patient selection and does not mean this was inevitable. While 30-day mortality was 100% in non-hospitalized patients and 83% in hospitalized patients, the same study showed that it was only 25% for those patients who were managed operatively [4]. The implication of this is that although a nonoperative approach does not seek to hasten a patient’s death, this may be one consequence of a focus on the comfort and dignity that is described in these papers.

## How do we approach the discussion of these 2 management options?

In the context of a patient with severe dementia any decision-making process is complex, and the ethical and legal framework within which this would happen will vary between countries.

If the patient had previously recorded their wishes, then this might prove useful, but in most countries such decision-making will not specifically include the care of an acute injury such as a hip or other fracture. The new study might help to inform future discussion of nonoperative hip fracture care, but it seems unlikely that many patients will have defined their future wishes in such detail.

If the patient has appointed a specific relative or friend to make decisions on their behalf, then some countries may permit them to decide on operative or nonoperative management. But before doing so they would need to be fully informed about the very different expectations of short- and longer-term survival we have mentioned.

If the patient has not made such plans for their future, it is difficult to envisage family, carers, and clinical staff being confident enough to select nonoperative end-of-life care as opposed to the prompt “palliative surgery” to reduce pain and allow patients to move with comfort and dignity during nursing care, which is now routine and so successful in developed countries. The ethical and psychological complexity of such decision-making is beautifully described in the paper by Rogmark and Lynøe [5].

## Implications for hospital care of these and other cases

Much of recent success in improving hip fracture care has turned upon a move away from the pessimism of previous decades, when patients, families, and staff had seen how poorly others had fared after a hip fracture. Nonoperative approaches were taken, and the likelihood of early mortality in such cases reinforced surgeons’ and anesthetists’ conviction that this was the right choice. Collaborative orthopedic-geriatric care combined with an understanding of the benefits of prompt surgery has moved things on, to a point where in the UK all but 3% of patients now have surgery, and mortality has improved as a result.

In this context, urgent surgery is the team’s assumption when a patient presents with a hip fracture, and the patient is immediately worked-up and consented for an operation. The sense of urgency of the whole pathway might be delayed if staff are unclear when a nonoperative approach might need to be considered.

Discussion of nonoperative end-of-life care with the family of a confused, previously very dependent patient is very complex, and tends to be poorly received if they have already been told of the urgency of surgery. Such conversations are not resource neutral as they may need to take up more senior clinical time than the operation would have done.

## Implications for palliative care in nursing home or hospital

The palliative care of someone with a hip fracture is very different from that of many other types of pain. A hip fracture causes severe, but very short-lived “incident pain” when the patient is moved, and non-specialist staff will not be familiar with how this might be handled.

This can make it difficult to nurse the patient with dignity. The work from the Netherlands has been important in its exploration of nonoperative options for the control of pain and other symptoms following hip fracture. The role of anesthetic or phenol nerve blocks deserves wider consideration and will be very relevant to the hospital care of cognitively intact patients who cannot have surgery.

At present such specialist end-of-life interventions would be very difficult to provide to patients in nursing homes in many countries. This would not currently be practical in the UK, though it might be possible with cooperation between hospital and nursing homes in the Nordic countries.

The study by Zeelenberg et al. has demonstrated high quality of symptom control in a group of very frail patients, providing high levels of satisfaction with treatment and quality-of-dying for those close to them [1]. The study is therefore to be welcomed as it questions the current treatment paradigm that a hip fracture must inevitably lead to a patient being taken to hospital. A study like this makes us stop and think, and is a timely reminder that we must all continuously reassess our treatment and care strategies for this patient group.

Complete disclosure of interest forms according to ICMJE are available on the article page, doi: 10.2340/17453674.2025.43190
